# Characterization and Modeling of Ply/Tool and Ply/Ply Slippage Phenomena of Unidirectional Polycarbonate CF Tapes

**DOI:** 10.3390/polym15173520

**Published:** 2023-08-23

**Authors:** Andreas Kapshammer, Daniel Laresser, Matei C. Miron, Felix Baudach, Zoltan Major

**Affiliations:** 1 Institute of Polymer Product Engineering, Johannes Kepler University Linz, Altenberger Str. 69, 4040 Linz, Austria; zoltan.major@jku.at; 2 Competence Center CHASE GmbH, Hafenstraße 47–51, 4020 Linz, Austria; daniel.laresser@chasecenter.at (D.L.); matei.miron@chasecenter.at (M.C.M.); 3 Covestro Deutschland AG, B207, R428, 51365 Leverkusen, Germany; felix.baudach@covestro.com

**Keywords:** ply/ply slippage, ply/tool slippage, high temperature, cohesive zone modeling, friction, thermoplastic composites, unidirectional tapes

## Abstract

Thermoplastic tapes are commonly processed by the rapid and efficient stamp forming process. During this forming process, the individual unidirectional tapes of the composite stack move relative to each other and relative to the surface of the tool while being in contact with the corresponding counterpart. As a result, the material exhibits a certain resistance against this movement, which is generally dependent on velocity, normal pressure, and temperature. Therefore, this work investigates the ply/tool and ply/ply slippage of unidirectional, carbon fiber reinforced polycarbonate tapes and provides an alternative implementation of the experimentally observed slippage using cohesive zone modeling. The backbone of the modeling approach is an experimental data set obtained from pull-through experiments. In comparison to common slippage or friction theories, the force plateau of thermoplastic UD tapes at elevated temperatures is observed after an initial force peak has been overcome. For both configurations, ply/tool and ply/ply, a reduction of the initial force peak was observed for increasing temperature. Furthermore, the resulting plateau force value is at least 36% higher in the ply/ply configuration compared to the ply/tool configuration at 200 °C. The derived cohesive zone model allows for accurate modeling of the initial force peak and the plateau.

## 1. Introduction

A rising attention in the field of composites is currently paid to thermoplastic based composites. The advantages and disadvantages of this materials class compared to its thermoset based counterparts were already part of scientific discussions over the last decades [1,2,3,4]. Stamp forming processes are very common in industrial large-scale productions of thermoplastic composites due to their simplicity and high throughput. Hereby, a flat two-dimensional preconsolidated composite blank, consisting of thermoplastic tapes, is formed into the desired shape by applying temperature and pressure [5]. The rising demand for this material class comes with an increased need for accurate simulation models that can complement experimental investigations, facilitate early defect identification and prediction, and thus enable cost- and resource-efficient manufacturing [5,6,7,8,9]. Heated thermoplastic tapes are heterogenous systems consisting of reinforcing fibers in the solid state surrounded by a molten thermoplastic polymer matrix. Thus, a superposition of effects related to solid and fluid mechanics can be observed at elevated temperatures [2,5,9,10]. During the forming process, a thermoplastic composite blank experiences different deformation mechanisms related to intra- and/or inter-ply properties [2,9,11,12]. In total, the resulting deformation of the laminate is a delicate balance between all deformations. Three dominant mechanisms have been identified, namely ply bending, inter-ply slippage (ply/tool and ply/ply) and intra-ply shear [12,13]. Inter-ply slippage has been investigated in different studies over the last three decades. One of the first experimental approaches, conducted in the 1990s, applied rotational rheometry methods on consolidated stacks [14]. In the following years, dedicated testing devices for continuous fiber reinforced tapes were developed and first approaches for modeling the experimentally observed behavior were derived [15,16,17]. A main finding discovered in the literature was the characteristic force-displacement curve derived from pull-through and pull-out measurements [18]. The work of Sachs et al. [9,12] and Vanclooster [10] investigated the influence of specimen temperature, normal pressure and pulling velocity. Expanding on the experimental results, different modeling approaches were investigated, such as phenomenological models based on modeling the material’s shear response or models derived from a Hersey number representation. Current numerical implementations utilize specially designed user subroutines or the Hersey number approach to implement slippage behavior [2,9,12,18,19,20,21]. The latter couples a numerically fitted coefficient of friction (CoF) with the so-called Hersey number, He, which is defined as lubrication parameter known from the tribological considerations [2,9,12,21,22,23,24]. It is defined as:

(1) $$He=\frac{\eta \left(T\right)\;v}{p_{N,l}}$$

η represents the temperature dependent viscosity of the material, v is defined as the sliding velocity and $p_{N,l}$ is the acting normal load per contact length. On the basis of the shown equation, the CoF can be calculated based on a parameter optimization of experimentally observed behavior [2,9,12,21,22,23,24]. These conventional friction models based on a static or dynamic CoF are able to predict slippage accurately.

However, they often fail in forming simulations due to neglecting the additional resistance to ply/tool (PT) or ply/ply (PP) separation emerging from the inherent tackiness of a molten polymer [25,26,27]. This property must be accounted for to prevent unrealistic ply separation during the forming process. Remarkably, despite its impact on simulation accuracy, the numerical modelling of the cohesive ply separation has been overlooked in previous implementations.

**Figure 1:**
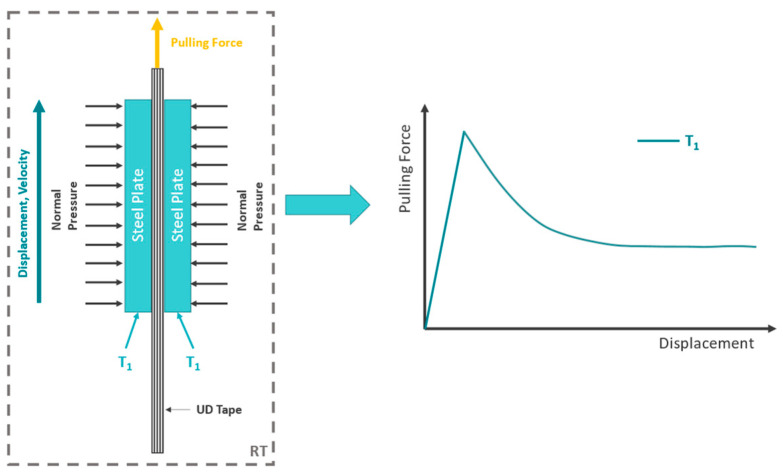
Schematic representation of a pull-through test setup for measuring ply/tool slippage properties (**left**) and a schematic plot of the resulting force-displacement curve at temperature T_1_ (**right**) indicating the force overshoot at the beginning and the following plateau region.

A main finding stated in literature and also observed in this study is the reproduceable shape of the experimentally determined force-displacement curve for different thermoplastic matrix systems when a single tape is pulled through or pulled out of a certain testing setup. Usually, the experimental setup consists of a specimen clamped between two heated metal plates (ply/tool) or additional tapes fixed on heated metal plates (ply/ply) by applying a defined normal load. After thermal equilibrium is reached, the specimen in molten matrix state is pulled through the testing device with constant velocity. The majority of the recorded force-displacement curves show an initial linear region followed by a peak and a subsequent decrease toward a plateau value as it is shown in Figure 1 [9,10,13,15,16,22,23]. A conceptual illustration of a pull-through test setup for ply/tool measurements is shown on the left. Amplification and mitigation of the initial peak as well as the steady-state level are strongly dependent on temperature, normal pressure, and pulling velocity [2,10,11,12,13,15,18,22,23].

**Figure 2:**
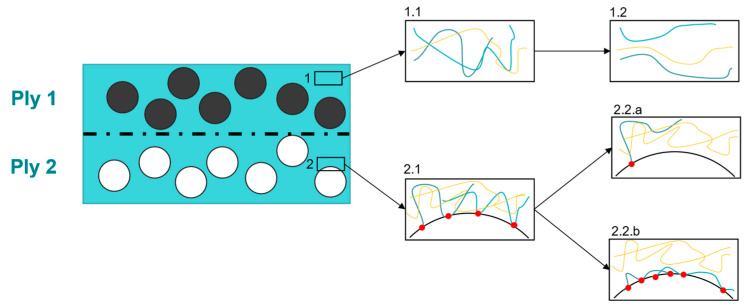
Cross section of two consolidated UD plies. Path 1 represents the mechanisms due to the NLVE theory (1.1—entangled macromolecules, 1.2—disentangled macromolecules) whereas path 2 deals with slip relaxation effects (2.1—initially adsorbed and entangled macromolecules, 2.2.a—desorption of macromolecules, 2.2.b—disentanglement of adsorbed macromolecules). The black and white circles indicate the unidirectional fibers in ply 1 and ply 2, respectively. Furthermore, the different macromolecules are shown by the colored lines in the corresponding boxes. The red dots are referred to as adsorption points between macromolecules and fibers. Figures adapted from [11].

The reason for the indicated force peak at the beginning is still not fully solved and is vividly debated in the literature, especially for ply/ply configurations [9,11,12,18,19,20,23,28,29]. At the moment, two different and competing theories exist [11]. A first explanation is derived from the inherent molecular structure of amorphous and semicrystalline polymers and refers to the entangled polymer chains, interacting with each other, based on nonlinear viscoelasticity (NLVE) [30,31]. It predicts that, up to a certain deformation rate, the polymer responds to the applied deformation by internal relaxation mechanisms leading to a disentanglement of the polymer chain network (see boxes 1.1 and 1.2 of Figure 2). For higher deformation rates, the junctions formed by these entanglements cannot be resolved in time and lead to the linear response until sliding occurs or the network is damaged, which could explain the observed force-displacement curves [10,11,30,31]. A concurrent theory attributes the presence of the peak to wall slip relaxation processes where matrix-fiber and matrix-wall interactions are considered [9,11,32]. This theory distinguishes between the desorption of polymer chains at the wall/fiber (box 2.2.a in Figure 2) and the disentanglement of already adsorbed macromolecules from the matrix (box 2.2.b in Figure 2) [9,11,12,32,33,34]. A representation of the basic principles according to both mentioned theories is depicted in Figure 2.

This study details the experimental characterization procedure of the inter-ply slip properties of unidirectional, carbon fiber reinforced polycarbonate tapes within the temperature range of stamp forming. Both ply/tool (PT) and ply/ply (PP) configurations were analyzed considering two different normal pressures and pulling velocities. The experimental results were analyzed in terms of temperature, normal pressure, and velocity dependency. In addition, a novel numerical modeling approach was developed utilizing a well-known traction-separation model to implement a correct representation of the inter-ply slippage behavior, enabling transient thermo-mechanically coupled forming simulations.

## 2. Materials and Methods

In the following section information about the investigated tape material is provided, followed by an explanation of experimental methodology and setup, which the physical part of this study is based on.

### 2.1. Used Materials

The investigated UD tape material Maezio^®^ (TACF170—44GP 1003T) consists of polycarbonate (PC) as matrix materials and continuous carbon fibers (CF) as reinforcing phase provided by the company Covestro AG (Leverkusen, Germany). Specimens were cut out from a 200 mm wide spool of wound-up tape.

### 2.2. Experimental Setup and Testing Conditions

**Figure 3:**
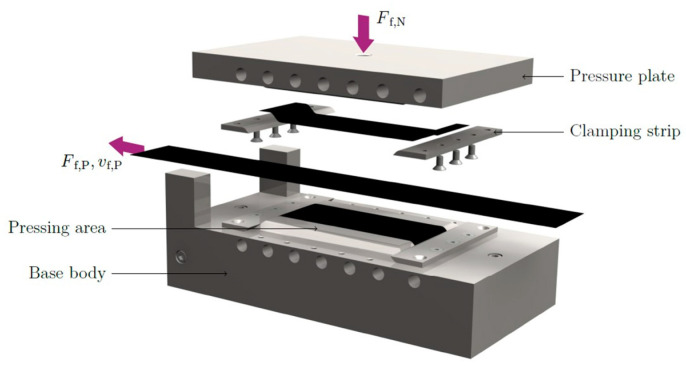
CAD rendering of the test setup used, reprinted from [2]. The figure illustrates the acting direction of the pulling force, $F_{f,P}$, pulling velocity, $v_{f,P}$ and the applied normal force, $F_{f,N}$.

The setup utilized to perform the physical pull-through experiments is based on the work of [2]. The specimen is clamped and placed between a base body and a pressure plate. Both consist of steel and were heated up to elevated temperatures by using four heating cartridges each, positioned close to the pressure face of the plates. A schematic representation of the setup, initially developed by [2], is shown in Figure 3 illustrating the acting forces and the corresponding tape positions.

**Figure 4:**
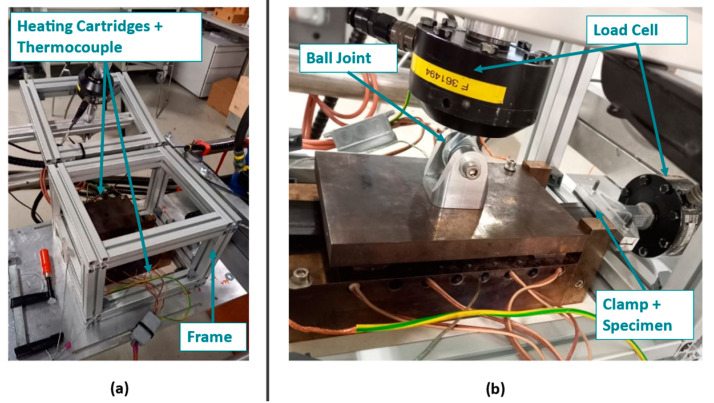
Utilized setup to perform the pull through experiments at elevated temperatures including all adjustments compared to the original one of [2]. (**a**) shows the general setup including the aluminum frame as well as heating cartridges and thermocouples for temperature control. (**b**) provides the position of the used load cells used for data recording including the initial specimen position and the ball joint to avoid tilting of the pressure plate.

For control purposes, a thermocouple was inserted in each of the base body and pressure plate, at the same distance from the pressure area. Responding to the recorded thermocouple signal a PID temperature control loop was implemented in LabView14 software (National Instruments, Austin, TX, USA). Thus, it was possible to adjust and control both plates separately. Further improvements on the testing rig in terms of normal force application were made compared to the original design. Through a threaded rod, supported by an aluminum frame, it is possible to define and record the normal force during the measurement via an additional load cell. In avoidance of any tilting of the pressure plate, a ball joint is placed between the load cell and the plate surface. As a testing machine, the hydraulic MTS 852 Test Damper System (MTS Systems Corporation, Eden Prairie, MN, USA) was used. Recording the pulling and normal force was performed using two 10 kN load cells. Figure 4 provides on the left-hand side (a) an illustration of the general setup including the aluminum frame, heating cartridges and thermocouples. In (b) a more detailed image is shown providing the position of the ball joint, load cells for recording pulling and normal force, as well as the clamped specimen.

**Figure 5:**
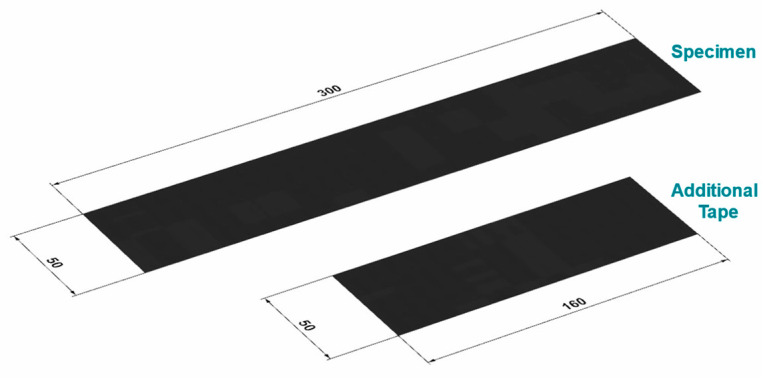
In-plane specimen and additional tape dimensions where the carbon fibers are aligned in longitudinal direction. The thickness of 0.175 mm is equal for both configurations.

For testing the ply/tool and ply/ply behavior, each specimen consisted out of one ply, having a thickness of 0.175 mm and in-plane dimensions of 300 × 50 mm^2^. The fiber orientation was colinear with the pulling direction in all performed experiments. To avoid matrix residues and ensure repeatable test conditions, the tool surfaces were cleaned after each measurement using a brass brush and isopropanol. In terms of PT configurations, the specimen was placed between the two cleaned and heated surfaces of the testing rig. Subsequently, the normal pressure was applied, and a time of 30 s was waited to ensure a homogeneous temperature distribution within the setup. The pull-through loading of the specimen was realized by a displacement-controlled procedure with different velocities and an end level of 60 mm. In the case of PP experiments, a similar workflow was deployed. Additionally, smaller tape sheets with a length of 160 mm were clamped to each pressing area of the testing rig, as illustrated in Figure 3. These additional tape sheets revealed an equal width and thickness as the specimen and were oriented with the same fiber orientation. A schematic representation of specimen and additional tape sheets is illustrated in Figure 5.

As already mentioned in the Section 1 of the study, two velocities and two normal pressures were applied. Concerning the temperature, three different values, in the forming temperature range, at 200 °C, 250 °C, and 300 °C, were applied for the for the experimental setups for PT and PP. Furthermore, an additional set was carried out at 100 °C for the PT configuration to get deeper insides in the sliding behavior of the polycarbonate material below its glass transition temperature. Each temperature- pressure-velocity configuration was carried out three times, leading to a total number of 84 experiments. A list that includes all configurations at the different temperatures for the PT and PP experiments is provided in Table 1. For comparison and evaluation purposes, the initial peak value of the pulling force and the mean force of the plateau region will be used. Whereas the latter one is calculated as the average force between the axial displacement of 30 mm to 45 mm.

## 3. Numerical Modeling

The aim of this work was the development of a numerical model to predict the slippage behavior of thermoplastic tapes within the stack itself and the interaction between tool surface and ply stack during stamp forming processes.

Classically, different friction models, based on static and dynamic friction coefficients, are available to model the sliding behavior observed for PT and PP interactions during forming. A drawback of these modeling approaches is the lack of ability to implement the out-of-plane interactions between individual plies. This out-of-plane interaction is necessary to model the adhesion between the plies in a state of already softened matrix material at elevated temperatures. Thus, the use of static or dynamic friction coefficients introduces an artificial degree of freedom for ply separation that is covered by ply adhesion in real world experiments. For this reason, another formulation has come to the forefront for describing the PT and PP interactions in thermoplastic composites. From a purely qualitative perspective, the characteristic force-displacement curves of pull-through experiments, performed with thermoplastic tapes in the forming temperature range reveals similarities with traction-separation curves of cohesive zone models from the literature [35]. Therefore, it is reasonable to fit the PT and PP behavior by using already known models similar to cohesive zone modeling. The main advantages are the wide distribution and implementation of such models, in commercial finite element software packages, which eliminates the need for user materials/subroutines and the problem-free application in explicit and implicit solver environments. In this study the multipurpose solver Abaqus (Simulia-Dassault Systemes, Providence, RI, USA) is used to implement and investigate the approach concerning cohesive zone modeling.

In the following subsection, the modeling of the PT and PP slippage behavior will be introduced based on a purely traction-separation formulation.

### 3.1. Cohesive Zone Modeling

**Figure 6:**
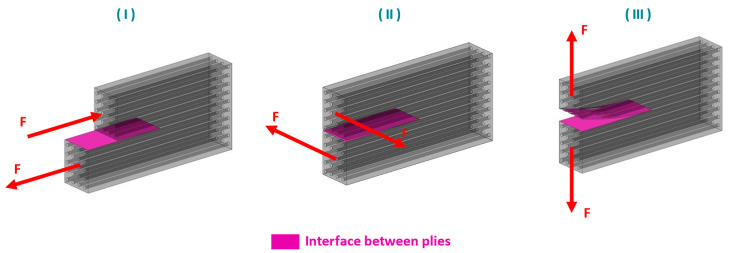
Schematic representation of the basic inter-ply movements within a thermoplastic UD ply stack. (**I**,**II**) indicate relative in-plane motions of two plies along and perpendicular to the fiber direction, respectively. (**III**) provides a schematic illustration of the ply separation (out-of-plane) motion.

In general, the slippage behavior between a composite stack and a forming tool, respectively between single composite plies can be divided into two relative in-plane (I and II in Figure 6) and one out-of-plane motions (III in Figure 6). A schematic representation of the mentioned basic inter-ply motions is shown in Figure 6.

**Figure 7:**
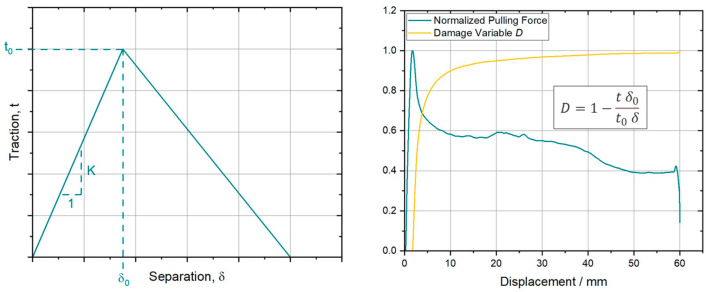
(**left**)—Schematic representation of a linear cohesive zone, traction-separation model (adapted from [29]); (**right**)—Representative normalized force—displacement curve.

For modeling purposes, a decoupled tabular formulation was chosen regarding the damage evolution of the nominal tractions in I direction, $t_{s}$, II direction, $t_{t}$, and III direction, $t_{n}$, was chosen. Based on the known contact area and the actual pulling displacement a conversion from the experimentally obtained pulling force over displacement relations to traction-separation, t−δ, curves can be performed. The workflow for was chosen according to the related software documentation [35]. To implement the observed softening behavior, a damage variable, D, is introduced. D can be calculated by utilizing the following Equation (2) [35,36].

(2) $$D=1-\frac{t\;\delta_{0}}{t_{0}\;\delta }$$

where $t_{0}$ and $\delta_{0}$ are describing the traction respectively separation at damage onset. The initial slope of the t−δ curve is related to the corresponding stiffness coefficients, $K_{ii}$, of the cohesive zone for i=s,t,n. Due to simplicity, it was assumed that the properties according to I and II are equal. This simplification is valid by neglecting fiber-fiber interactions (no influence of fiber orientation in different plies) and by assuming an isotropic polymer melt film or interface for PT and PP, respectively. As a result, the derivation of the modeling parameters regarding I and II direction can be realized by the experimental data determined by only one experimental pull-through configuration, as introduced in Section 2.2. Furthermore, the cohesive zone approach provides the option to model ply separation by calibrating the corresponding traction-separation properties in III direction. This is not within the scope of this work but a crucial requirement for the application of the current model, in full-scale forming simulations. Figure 7 shows on the left-hand side a schematic representation of a common bilinear traction separation formulation including the mentioned parameters. On the right-hand side of this figure the underlying damage modeling based on Equation (2) is shown on the example of a representative, normalized force–displacement curve. With this approach, the ply/tool or ply/ply behavior will be modeled only by the damage variable over the full displacement scale up to 60 mm relative displacement.

## 4. Results and Discussion

This section provides all results concerning the experimental evaluation of the PT and PP slippage behavior of thermoplastic PC UD tapes, reinforced with continuous carbon fibers. In particular, the temperature, pressure, and pulling velocity dependency will be analyzed. Further, a comparison of the experimental and numerical results will be provided and discussed, to investigate the model parameters derived in Section 3.1.

### 4.1. Experimental Results

#### 4.1.1. Ply/Tool Experiments

**Figure 8:**
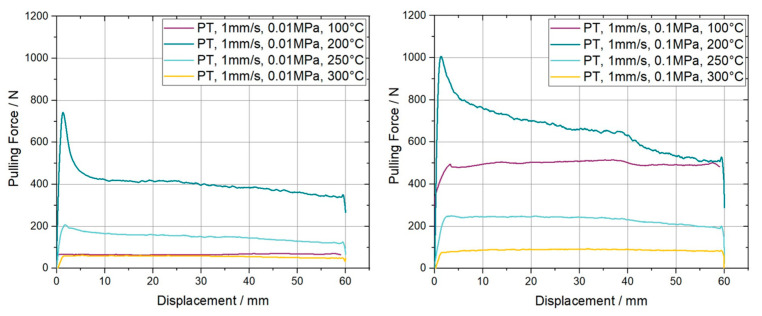
Resulting pulling force-displacement curves for ply/tool pull through slippage experiments on carbon fiber reinforced PC UD tapes. The curves are measured at a pulling velocity of 1 mm/s, two different normal pressures and four temperatures.

As previously introduced the ply/tool slippage behavior was experimentally determined at four different temperatures. Three were chosen in the forming temperature range between 200 °C and 300 °C and one was selected below T_g_ of the material, at 100 °C. The two plots in Figure 8 represent the derived pulling force over displacement for the different temperatures. On the left-hand side, the plot shows the results accomplished at a normal pressure of 0.01 MPa and on the right-hand side the corresponding line plots for the experiments at 0.1 MPa are presented.

**Figure 9:**
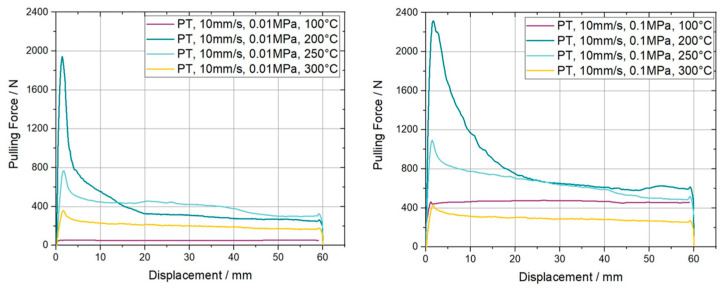
Resulting pulling force-displacement curves for ply/tool pull-through slippage experiments on carbon fiber reinforced PC UD tapes. The curves are measured at a pulling velocity of 10 mm/s, two different normal pressures and four temperatures.

The characteristic shape of the curves including a force peak at the beginning, followed by a leveling off at a certain plateau region, can be clearly observed for 200 °C, indicated by the dark blue line, for both normal pressures. The peak value as well as the plateau level is 36% and 63% higher, respectively, for a normal pressure of 0.1 MPa compared to the lower one of 0.01 MPa. For increasing temperatures, the initial overshooting effect started to decrease until it was no longer present for a temperature of 300 °C. Also, the plateau values are reducing from 390 N at 200 °C to 60 N at 300 °C for elevated temperatures at a velocity of 1 mm/s. An analogous trend can be found for the curves at higher normal pressure. Towards the final displacement of 60 mm the provided curves in Figure 8 show a slight increase in force followed by a rapid decrease in force until the measurement is stopped. This behavior is induced by a change in the measurement principle due to the large displacement, from a pull-through to a pull-out measurement. A similar behavior is visible for all experimental results presented in this study. The measured data at 100 °C reveals a behavior similar to Coulomb’s friction, where the resulting pulling force is dependent on the normal pressure. This can be stated because the values are equal to those obtained with a pulling velocity of 10 mm/s at the corresponding pressures. Figure 9 provides the related curves in an analogues line plot.

**Figure 10:**
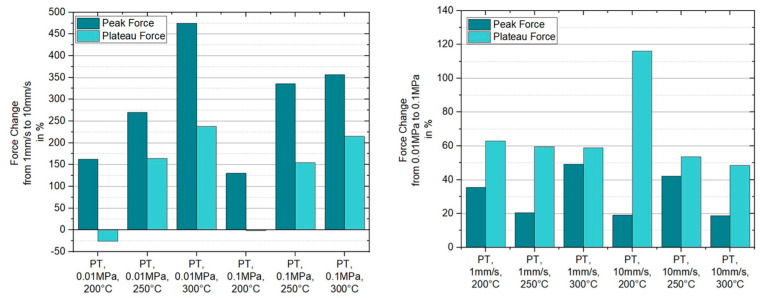
Percentual changes of the initial peak and plateau force in respective to a change of pulling velocity and applied normal pressure in the case of ply/tool slippage.

For a pulling velocity of 10 mm/s one can observe a more pronounced presence of the initial force overshooting compared to the results at 1 mm/s, for all temperatures in the forming range. Comparing the results for 200 °C, 1 mm/s and 200 °C, 10 mm/s at 0.01 MPa, the initial peak value of the latter set is 2.6 times higher than the former. An increase of 270% and 475% for a ten times higher pulling velocity can be stated for the two remaining temperatures at 250 °C and 300 °C. A similar trend is shown for the plateau values. Noteworthy is the behavior of the plateaus at the higher pulling velocity for 200 °C and 250 °C, revealing a converging force development. Hereby, the force drop at 200 °C is much higher than the one at 250 °C. The influence of the normal pressure is also present for higher loading rates by pronouncing a similar change of the total numbers as was stated for the experiments at 1 mm/s. Another interesting finding is the significance of the initial force overshoot for higher temperatures at 10 mm/s versus the vanishing of the peak at the lower velocity. This observation and the in total higher influence of the loading rate/pulling velocity, see Figure 10, strengthens the assumption that the observed characteristics are related to the NLVE. Figure 10 presents in a compact format the derived percentual changes of the initial peak and plateau force dependent on the pulling velocity and the applied normal pressure. For curves without a peak, the slope transition point, where the slope changed its sign or reduces below 25% of the initial slope value, was used as a reference for the peak force.

Since the influence of temperature, pulling velocity and normal pressure was characterized through the performed pull-through experiments, an analysis of variance (ANOVA) was conducted to determine the significance of the mentioned parameters. Therefore, a single factor ANOVA was performed for each parameter configuration with respect to an alpha-value of 0.05. The peak and plateau force were investigated separately regarding the significance analysis. As a result, it can be stated that temperature, pulling velocity, and applied normal pressure have a significant influence on the peak and plateau force in ply/tool slippage configurations.

#### 4.1.2. Ply/Ply Experiments

**Figure 11:**
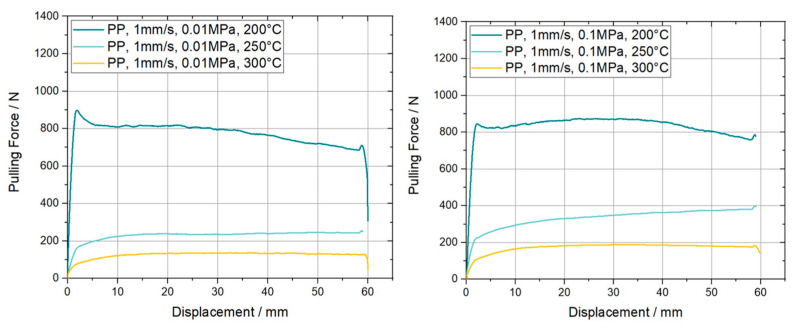
Resulting pulling force-displacement curves for ply/ply pull-through slippage experiments on carbon fiber reinforced PC UD tapes. The curves are measured at a pulling velocity of 1 mm/s, two different normal pressures and three temperatures.

**Figure 12:**
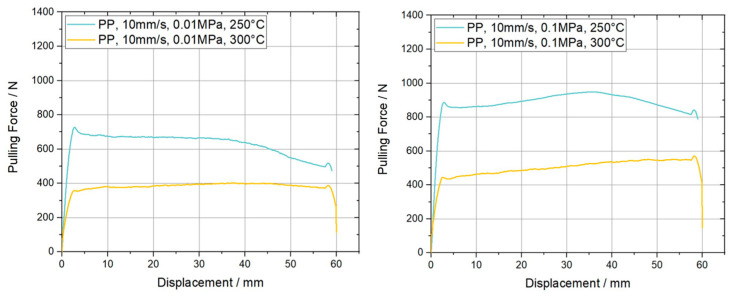
Resulting pulling force-displacement curves for ply/ply pull-through slippage experiments on carbon fiber reinforced PC UD tapes. The curves are measured at a pulling velocity of 10 mm/s, two different normal pressures and temperatures.

**Figure 13:**
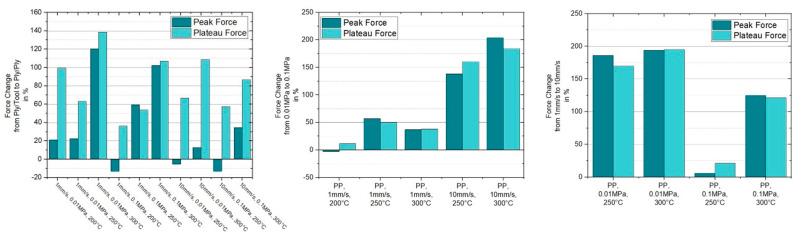
Percentual changes of the initial peak and plateau force in respective to a change of configuration from ply/tool to ply/ply (**left**), pulling velocity (**center**) and applied normal pressure (**right**).

The characterization of the ply/ply slippage was elaborated only above T_g_ of the PC-CF UD tape, again at 200 °C, 250 °C and 300 °C, for equal sets of normal pressure and pulling velocity. Furthermore, the PP experiments were carried out on a [0°, 0°, 0°] stacking sequence, which means that both additionally applied plies and the specimen were aligned similarly. Resulting the fiber direction was colinear to the pulling direction. Figure 11 presents the pulling force vs. displacement curves for the different PP configurations considering a pulling velocity of 1 mm/s. Compared to its equivalent counterparts in PT experiments the plateau force values are increased by at least 36% in case of 200 °C, 0.1 MPa and 1 mm/s. A significant mitigation of the initial force peak is also visible, leading to curves without any overshooting at all like those for 250 °C and 300 °C at high and low normal pressures. Another noteworthy aspect is an increase of the pulling force, in experiments with a normal pressure of 0.1 MPa, after the already mentioned start up effects, within the range where a constant value (plateau) should be formed. This effect is visible both at 1 mm/s and 10 mm/s pulling velocity and is already more present for the latter velocity. Interestingly, even a higher force was reached than compared to the initial maximum force for the settings PP-1 mm/s-0.1 MPa-200 °C and PP-10 mm/s-0.1 MPa-250 °C, while the corresponding plots for 10 mm/s are depicted in Figure 12. The reason for this effect was not investigated in this paper and will be part of future research. Again, an equivalent trend concerning temperature and normal pressure dependence can be observed as mentioned for the PT results. A visual representation of the indicated trends is depicted in the column plots of Figure 13.

Similar to the variance analysis introduced in Section 4.1.1, an ANOVA was conducted to investigate the significance of the three varied parameters. Again, a significant influence of temperature, pulling velocity and normal pressure was observed.

### 4.2. Numerical Results

This section will provide all fitted parameters as well as the results of the performed validation simulations for each modeling strategy.

**Figure 14:**
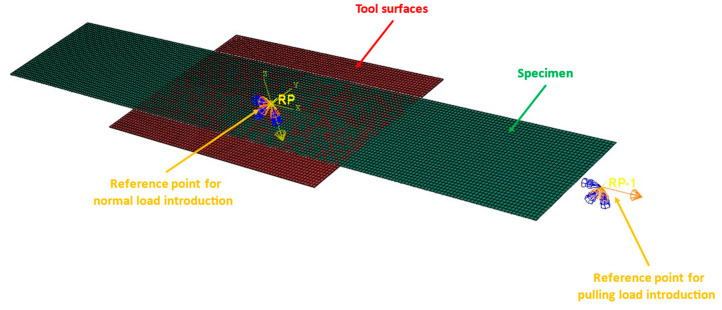
Illustration of the used CAE model for the numerical simulation of PT configurations. Similar setup for PP configuration by replacing tool surfaces with the corresponding additional ply geometries.

For calibration and validation purposes, a digital representation of the experimental slippage testing rig was modeled within Abaqus/CAE. Equivalent specimen and additional tape geometries were used to model the parts using reduced integrated, linear hexa-hedral elements (C3D8R) with a mesh size of 1.5 × 1.5 mm^2^ and two elements over the ply thickness of 0.175 mm. Resulting in a total number of 13,200 elements and 8480 elements for the specimen and additional tapes, respectively. In the case of a ply/tool configuration the steel surfaces were modelled by rigid quadrilateral shell elements (R3D4) with a mesh size of 1.5 × 1.5 mm^2^ and a total number of 4240. A cohesive contact formulation was utilized to implement the derived model parameters including damage initiation and damage evolution. For a convenient load introduction, two reference points were coupled with the corresponding surfaces of the model. One for applying normal pressure on the outer contact areas and another one for pulling the ply specimen through the testing rig via a certain velocity. For solving the numerical model, the Abaqus/Standard implicit solver was used. In Figure 14 the model for the PT simulations is illustrated.

Based on the modeling strategy introduced in Section 3.1, the stiffness values for the cohesive traction-separation model, $K_{ss}=K_{tt}$, the damage initiation separation $\delta_{0}$ and a table according to the evolution of the damage variable, D, was derived. For convenience and numerical stability, the stiffness value in mode I direction was chosen magnitudes higher than the in-plane parameters at a value of $K_{nn}=10^{6}\;N/{\mathrm{mm}}^{3}$. Through the linear interpolation between the entries of the damage table, it is possible to introduce artificial numerical instabilities which would lead to soaring effects. As a result, the calculated pulling force response can jump between the single entries of the damage table if fewer points are chosen for the modeling. According to the experimental results at different temperatures, it was possible to generate a set of temperature dependent modeling parameters to enable coupled thermomechanical simulations, in principle. The calibrated parameters for each temperature and an indication of the damage variable table are shown in Table 2, for the example of PT, 1 mm/s, 0.01 MPa.

**Figure 15:**
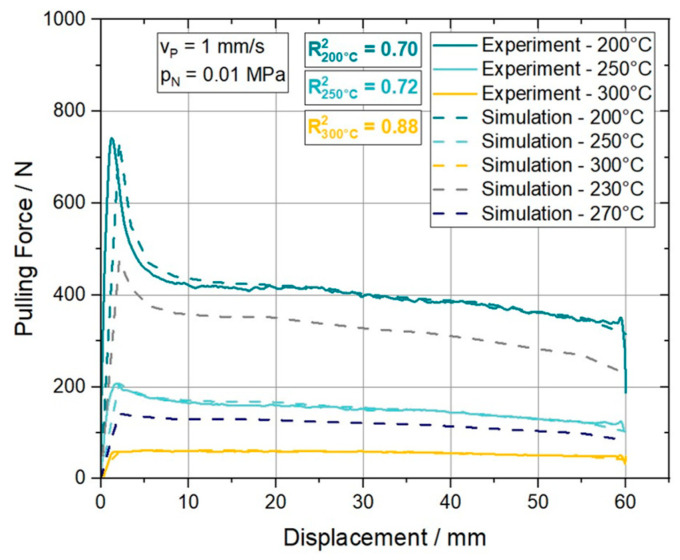
Validation of the traction-separation approach to model the ply/tool slippage behavior of the ply/tool. The curves provided represent the data set determined at a pulling velocity (v_p_) of 1 mm/s and a normal pressure (p_N_) of 0.01 MPa at different temperatures.

As a remark, the stated values for the $\delta_{ss}$ respectively $\delta_{tt}$, were determined within the calibration process applying a best fit procedure. By implication this explains the unsteady increase for the maximum separation parameter for higher temperatures. Further, a plausible reciprocal proportionality concerning the calculated stiffness values of the model and the temperature can be seen. To evaluate the accuracy of the model, a comparison between the experimentally and numerically derived pulling force over displacement curves is provided in Figure 15. Furthermore, the coefficient of determination ($R^{2}$) is considered as a statistical measure to evaluate the model fit by a quantitative value. In addition to the temperatures of the experimental trials, two more simulations were performed at 230 °C and 270 °C to verify whether a proper interpolation between the data sets is feasible.

It can be stated that the model fits the experimental curves well over the entire pulling distance. The initial response of the model at 200 °C and 250 °C is slightly more compliant than the measured response of the experiment. The plateau region is represented accurately at all temperatures. Furthermore, this traction separation approach allowed the authors to model curves including the characteristic initial force peak or curves without it properly. Through the simulations at temperatures in between (230 °C and 270 °C) of the modeling temperatures (200 °C, 250 °C and 300 °C) it is shown that a plausible interpolation in terms of temperature is possible. From a statistical perspective, the $R^{2}$ values for 200 °C and 250 °C are at 0.70 and 0.72, respectively. Whereas the prediction quality of the model at 300 °C can be quantified with $R^{2}=0.88$. With respect to all performed experiments the model performs within a $R^{2}$ range of 0.70 and 0.95 as it can be seen in Table 3.

## 5. Conclusions

In this work it has been attempted to analyze the ply/tool and ply/ply slippage behavior of continuous carbon fiber reinforced polycarbonate tapes funded on experimentally determined data. Furthermore, a novel modeling approach utilizing cohesive zone modeling was introduced to implement the corresponding ply slippage behavior into commercial FE solver.

The experiments were performed on 200 °C, 250 °C and 300 °C, at normal pressures of 0.01 MPa and 0.1 MPa for two different pulling velocities of 1 mm/s and 10 mm/s. Both the PT and PP results, presented in Section 4.1.1, reveal from a qualitative point of view a good agreement with the characteristic force-displacement curves stated in literature. Further, the force peak at the beginning is not present by performing this type of experiment below the glass transition temperature of the thermoplastic polymer matrix. For an increasing temperature, within the forming temperature range, a reduction of the initial force peak can be stated, as shown in the figures of Section 4.1 and Section 4.2. Analogously, the absolute force level over the whole measurement length shows a reduction at elevated temperatures, whereas the opposite trend is observed for increasing normal pressure and pulling velocity values. However, a change from PT to PP configuration results in average, in 34% higher peak load levels and 82% higher plateau forces needed for an equivalent relative displacement. A more significant influence was found for increasing velocities and pressure, which lead to an average peak force as well as plateau force increase of up to 200%. In total, the significant influence of temperature, pulling velocity and normal pressure has been proven by conducting the corresponding single factor analysis of variance for each investigated configuration.

The pronounced temperature and pulling velocity dependency of the force peak at the beginning emphasizes the hypothesis that this phenomenon is related to the nonlinear viscoelastic response of the entangled polymer chains. Furthermore, the reduction of the peak for higher temperatures and lower pulling velocities is plausible if the corresponding NLVE theory is considered. Since it is not possible to draw detailed inferences from the macroscopic pull-through experiments about the polymer chain motion, further research is needed to understand the complex ply/tool and ply/ply slippage behavior of thermoplastic tapes in detail.

Numerical modeling based on the principles of cohesive zone modeling provides good results. This approach enables the description of the initial peak of the ply/tool and ply/ply interactions, together with the following plateau region, within a single model. Furthermore, the cohesive model is applicable for cohesive contact and cohesive element formulation whereby it can be applied in coupled thermal-mechanical simulations.

## Figures and Tables

**Table 1:** List of performed ply/tool and ply/ply experiments incl. set temperature, pulling velocity and applied normal pressure.

Ply-Tool Experiments		
Temperature/°C	Pulling Velocity/(mm/s)	Normal Pressure/MPa
100	1	0.01
100	1	0.1
100	10	0.01
100	10	0.1
200	1	0.01
200	1	0.1
200	10	0.01
200	10	0.1
250	1	0.01
250	1	0.1
250	10	0.01
250	10	0.1
300	1	0.01
300	1	0.1
300	10	0.01
300	10	0.1
**Ply-Ply Experiments**		
**Temperature/°C**	**Pulling Velocity/(mm/s)**	**Normal Pressure/MPa**
200	1	0.01
200	1	0.1
200	10	0.01
200	10	0.1
250	1	0.01
250	1	0.1
250	10	0.01
250	10	0.1
300	1	0.01
300	1	0.1
300	10	0.01
300	10	0.1

**Table 2:** Calibrated cohesive zone model parameters for the ply/tool setting at 1 mm/s pulling velocity and 0.01 MPa normal pressure. Including values concerning stiffness, damage initiation and damage evolution.

Stiffness—Traction-Separation (Uncoupled)			
$K_{nn}$/(N/mm^3^)	$K_{ss}$/(N/mm^3^)	$K_{tt}$/(N/mm^3^)	Temperature/°C
10^6^	0.098	0.098	200
10^6^	0.019	0.019	250
10^6^	0.0061	0.0061	300
**Damage Initiation—Maximum Separation**			
$\delta_{0,nn}$ **/mm**	$\delta_{0,ss}$ **/mm**	$\delta_{0,tt}$ **/mm**	**Temperature/°C**
10^6^	1.26	1.26	200
10^6^	1.80	1.80	250
10^6^	1.44	1.44	300
**Damage Evolution—Tabular**			
Damage Variable (D **)/-**	$\mathrm{Plastic}\;\mathrm{Displacement}\;(\delta -\delta_{0}$ **)/mm**		**Temperature/°C**
0	0		200
…	…		…
0.94	20		250
…	…		…
0.98	60		300

**Table 3:** Resulting coefficient of determination ($R^{2}$) of the cohesive zone model with respect to the ply/tool and ply/ply experiments.

Ply-Tool Experiments			
Temperature/°C	Pulling Velocity/(mm/s)	Normal Pressure/MPa	*R*^2^—Value/-
100	1	0.01	0.90
100	1	0.1	0.70
100	10	0.01	0.84
100	10	0.1	0.75
200	1	0.01	0.70
200	1	0.1	0.85
200	10	0.01	0.87
200	10	0.1	0.93
250	1	0.01	0.72
250	1	0.1	0.85
250	10	0.01	0.91
250	10	0.1	0.74
300	1	0.01	0.88
300	1	0.1	0.79
300	10	0.01	0.73
300	10	0.1	0.72
**Ply-Ply Experiments**			
**Temperature/°C**	**Pulling Velocity/(mm/s)**	**Normal Pressure/MPa**	***R*^2^—Value/-**
200	1	0.01	0.95
200	1	0.1	0.72
200	10	0.01	0.72
200	10	0.1	0.78
250	1	0.01	0.77
250	1	0.1	0.72
250	10	0.01	0.92
250	10	0.1	0.89
300	1	0.01	0.81
300	1	0.1	0.88
300	10	0.01	0.74
300	10	0.1	0.87

## Data Availability

Data is not publicly available.
